# Online Bridge Crack Monitoring with Smart Film

**DOI:** 10.1155/2013/303656

**Published:** 2013-12-30

**Authors:** Benniu Zhang, Shuliang Wang, Xingxing Li, Zhixiang Zhou, Xu Zhang, Guang Yang, Minfeng Qiu

**Affiliations:** Department of Information Science and Engineering, Chongqing Jiaotong University, Chongqing 400074, China

## Abstract

Smart film crack monitoring method, which can be used for detecting initiation, length, width, shape, location, and propagation of cracks on real bridges, is proposed. Firstly, the fabrication of the smart film is developed. Then the feasibility of the method is analyzed and verified by the mechanical sensing character of the smart film under the two conditions of normal strain and crack initiation. Meanwhile, the coupling interference between parallel enameled wires of the smart film is discussed, and then low-frequency detecting signal and the custom communication protocol are used to decrease interference. On this basis, crack monitoring system with smart film is designed, where the collected crack data is sent to the remote monitoring center and the cracks are simulated and recurred. Finally, the monitoring system is applied to six bridges, and the effects are discussed.

## 1. Introduction

Bridges play a significant role in the economic development for the conveniences to the traffic and transportation. However, owing to the complexity of the force condition as well as the uncertainty of the surrounding environment, lots of unstable factors have been forced to the bridge after the construction, such that it is difficult to grasp the mechanical prosperities and behaviors during the operation. The frequently collapsed accidents of bridges endanger social well-being and stabilization. In view of the importance of bridge security, bridge health monitoring, especially in the field of crack monitoring, has made a rapid progress and become a hot topic in the current research.

Crack is a macroreflection of deterioration and lesion of concrete structures. Due to crack propagation [1], secondary diseases can be caused, such as leakage, corrosion of steel bars, and concrete carbonization, further affecting normal usage and the safety of the structures.

The crack monitoring methods usually used for concrete bridges include point monitoring, distributed monitoring and image recognition monitoring method. The point monitoring method [2, 3] places sensors individually at certain critical points on the structures to monitor crack. It can only be feasible when the critical points are predicted accurately. Unfortunately, in engineering practice, cracks do not always appear on the critical points owing to material-heterogeneity and calculation errors.

Recently, distributed monitoring method, which based on the optical fiber sensor, has been introduced in structural crack monitoring [4–9]. It has the characteristics of large information transmission capacity, high transmission speed, and high sensitivity. However, brittle optical materials with the order of several meters in length and only micrometer in diameter are difficult to be glued on or embedded into structures. Meanwhile, coaxial cable distributed sensor with electrical time domain reflectometry (ETDR) technology is buried in structures to collect strain and crack signals [10–12]. Still, this method has potential defects in installation, reliability, and stability of signals.

High-resolution camera lens make it available to monitor cracks by collecting real-time images of structures [13–15]. This method has a higher feasibility for short-term crack monitoring in a given small area, but it has difficulty in monitoring tiny crack.

Smart film monitoring method is proposed by the authors from empirical analysis and engineering practice. Smart film, in which a great number of insulated enameled copper wires are embedded to form a coordinate grid, is glued on the surface of the structure. Electrical signal is sent from one terminal of each wire, and the signal at the other hand is simultaneously detected. When a crack initiates, the conducting wires will break correspondingly. Therefore the signals will not get through those broken wires. By analyzing the conducting and breaking wires, initiation, length, shape, propagation, and the location of cracks can be identified. At the same time, crack width can be monitored by using the corresponding relationship between the diameter and crack width [16–18].

**Figure 1:**
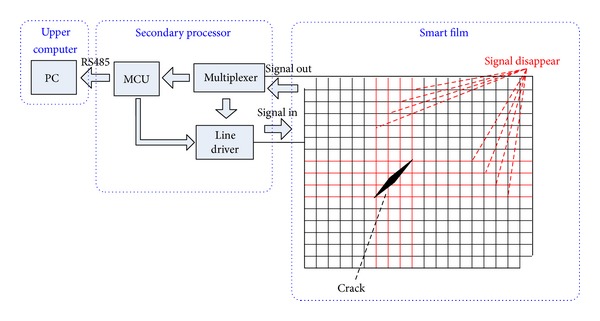
Schematic of smart film monitoring technique.

The crack sensing system built by this technique mainly consists of smart film, secondary processor, and upper computer. The smart film can be used for crack sensing. The secondary processor determines the location and the length of the crack according to the signal of the smart film and communicates with the upper computer for sending crack data. The upper computer processes and sends crack data to the remote monitoring center [16] (see Figure 1).

According to the above principles, the following problems may be encountered in the practical application. One of them is misjudgment of the crack owing to different reasons. Firstly, the smart film is glued on surface of the structure with epoxy resin and normal strain of the structure may break the enameled wire, thus resulting in misjudgment. Secondly tiny crack on the structure may not be detected because of no breakage of the wire. In addition the coupling effects between the wires can lead to the misjudgment, since all the wires of the smart film are parallel with each other and form a bunch of bus. Finally, the smart film, the secondary processor, and the upper computer can only be used for sensing and collecting the crack information. Therefore, to reach the intelligent monitoring of the concrete structure and accurate evaluation of the crack data, the system of data analysis and processing needs to be further developed.

**Figure 2:**
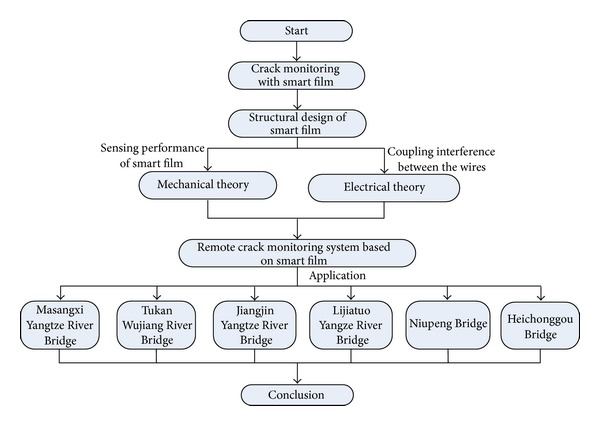
Diagram of the logic structure of this paper.

To deal with the problems discussed above, a methodological approach on bridge crack monitoring with smart film is developed in this paper. Processing techniques are considered and the logic structure is illustrated in Figure 2. Firstly, the fabrication of the smart film is firstly proposed for engineering practice. Secondly, by means of the mechanical sensing character of the smart film under the two conditions of normal strain and crack initiation, the feasibility of crack monitoring is analyzed and verified. At the same time, it is found that the enameled wires will not break under the normal strain condition, but when a crack passes through the wires, the wires will break accordingly. Then the phenomenon of coupling interference between parallel enameled wires of the smart film is revealed, and then low-frequency detecting signal and the custom communication protocol are adopted to reduce interference. On this basis, a crack monitoring system composed of the local monitoring center and the remote monitoring center is designed. The collected data of the local monitoring center is sent to the remote monitoring center, and then the crack will be simulated by the plug-ins of the interactive 3D scene and analysis of the crack data. Finally, the monitoring system is applied to six bridges including Masangxi Yangtze River Bridge and Tukan Wujiang River Bridge, and the effects are discussed. At the same time, the effectiveness of the results is testified by the comparison between the result of the man-operated periodic detection and that of monitoring.

This paper is structured as follows.  Section 2 describes the fabrication of the smart film in detail.  Section 3 analyzes the mechanical and electrical character of the smart film, attests the feasibility of crack monitoring under the two conditions of normal strain and crack initiation, and proposes the effective measures to prevent the signal interference between the wires. Then in Section 4, a description of the remote monitoring system used for the intelligent monitoring and the plug-ins of analysis and recurrence of crack data is given. Nextly, Section 5 discusses the applications of the remote crack monitoring system on six bridges including Masangxi Yangtze River Bridge. Finally, conclusions are presented in Section 6.

## 2. The Structural Design of Smart Film

**Figure 3:**
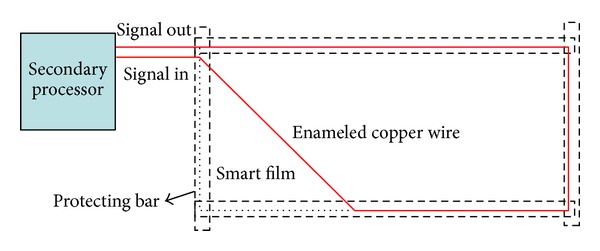
Road for one wire.

According to the crack monitoring technique based on smart film, enameled wires form the smart film regularly. Due to the small diameter of the wires, they are not easy to be fixed during the fabrication but easy to be broken. Therefore, plastic film with adhesive is used as substrate and glossy paper as the protection framework; then each wire is adhered on substrate as well as the bus protection framework and forms a grid as shown in Figure 3.

**Figure 4:**
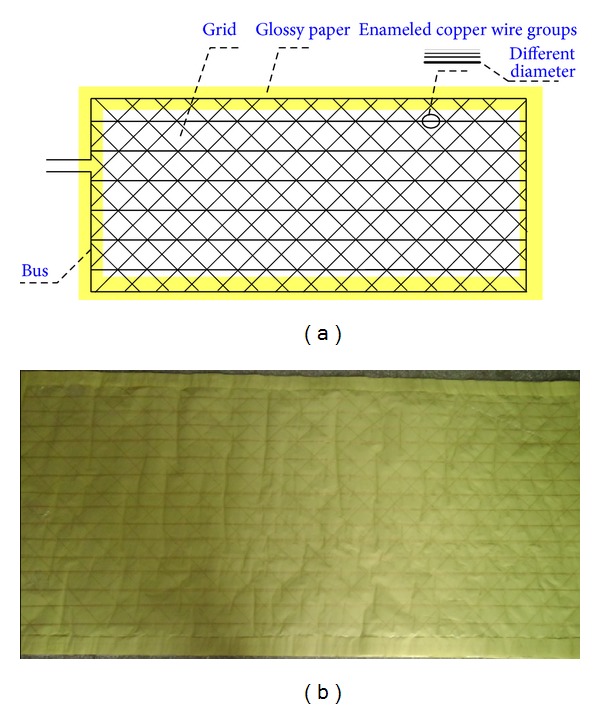
Smart film: (a) schematic and (b) photograph.

Different diameter has different ultimate strain. Therefore, a group of different diameter wires are placed transversely to monitor crack width. It is shown in Figure 4.

## 3. The Mechanical and Electrical Theory of the Enameled Wires

### 3.1. Mechanical Analysis [17]

Aimed to guarantee that the wires pasted on structures remain intact when no cracks appear on the structures, the wires must have larger failure strain than that of the concrete material. Here the ultimate strain of several kinds of enameled copper wire and epoxy are proofed and they are compared with the ultimate strain of common concrete material. According to the proof results, the ultimate strain of the wires is not less than 90000 *με*, and the strain of the epoxy resin is not less than 20000 *με*. Hence enameled copper wires will not be broken under normal strain conditions of concrete structure.

**Figure 5:**
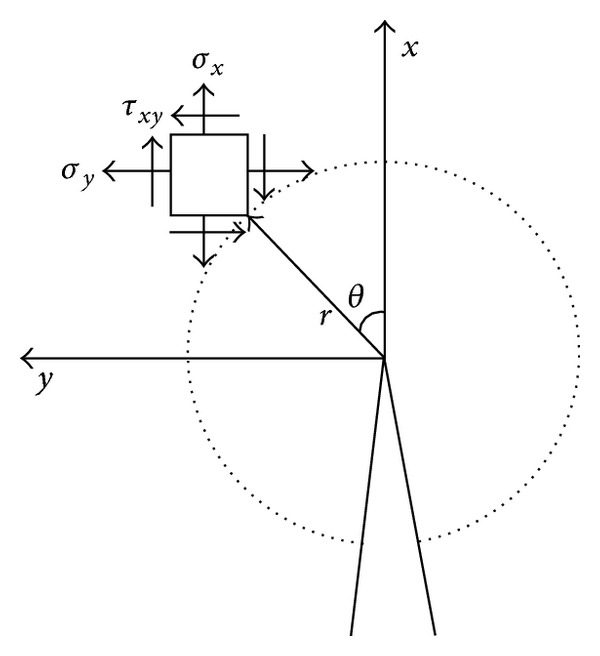
2D plane strain of a crack.

Considering that the structures being monitored are much larger than diameter of enameled wires, and the cracks will pierce through transverse direction of the wires, then the crack problem can be regarded as a 2D plane strain problem, as illustrated in Figure 5.

**Figure 6:**
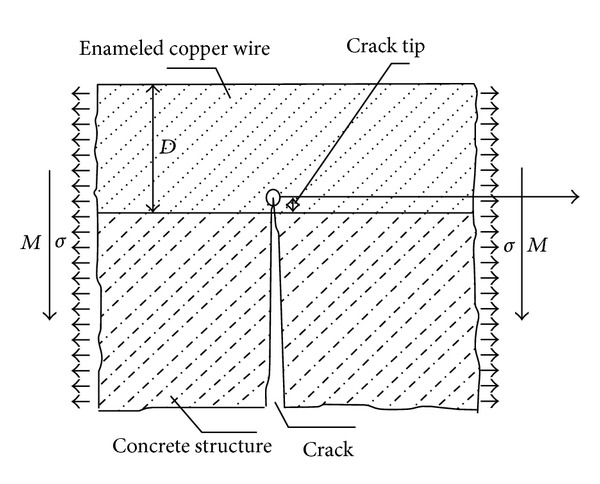
Concrete crack pierces to enameled wires.

Assume that the crack appearing on concrete structure has pierced enameled wire to generate a crack tip (Figure 6); the stress field in the vicinity of crack tip can be obtained. Then strain field nearby the crack tip (the singular) will be much greater than the ultimate strain. Hence, as long as the crack pierces the enameled copper wire, it will be broken [17].

### 3.2. Antiinterference Analysis

**Figure 7:**
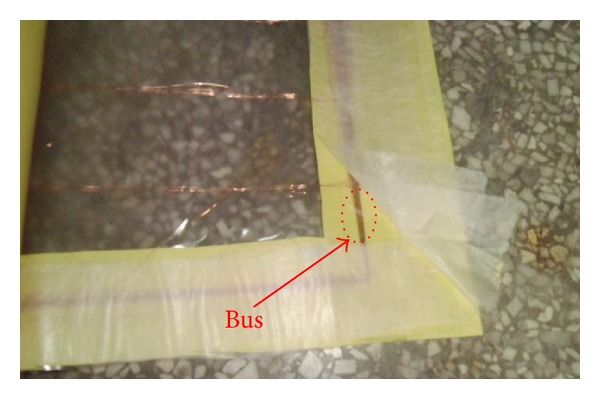
Bus of smart film.

It can be seen from the structure of smart film that enameled wires are forming parallel lines at regular intervals (see Figure 7). During the period of sending detecting signal to judge wires' condition, the voltage of interference coupling is so easy to be generated due to parasitic capacitance that conditions are judged wrongly. Hence it is necessary to analyze coupling interference among the bus of smart film and find the suitable detecting signals and detection ways, aimed to reduce coupling interference and enhance the accuracy of condition judgments of wires.

**Figure 8:**
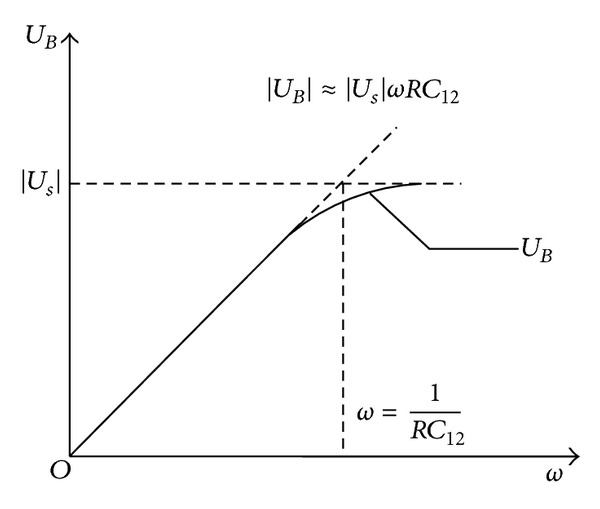
Relationship between coupling voltage and the frequency of detecting signal.

Selecting two wires from the bus in Figure 7, a model can be formulated to analyze and infer the relationship between the voltage ∪_*B*_ of coupling interference and the frequency *ω* of detecting signal, as demonstrated in Figure 8.

It can be seen from the figure that interference still exists. Only by lowering the frequency of detecting signal, coupling interference generated by parasitic capacitance can be effectively reduced. However, the speed of detection will be affected if the detecting frequency is very low. Therefore, 1 kHz is used as the frequency of the detecting signal in engineering practice. At the same time, in order to further improve the accuracy of condition judgments of the enameled wires, the custom communication protocol is also adopted. That is, after choosing a single wire, signal detection is sent 8 times circularly with high and low levels. Only when the levels are detected accurately by the output port, the enameled wire is thought to be intact.

## 4. The Remote Crack Monitoring System Based on Smart Film

**Figure 9:**
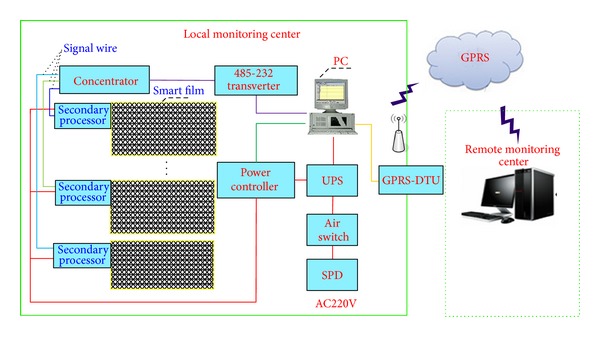
Hardware schematic of the remote crack monitoring system based on smart film.

In practical monitoring, the smart film can be only used for sensing of crack information; the secondary processor can collect and send the data to the upper computer. In order to monitor concrete structures intelligently, master the real-time and accurate crack information, and improve the readability of crack data, the data in the upper computer need to be sent to the remote monitoring center timely, accurately, and stably. The remote monitoring center will analyze and process the data to judge whether the crack will lead to damage of the structure. Therefore the remote system for transmitting, storing, and analyzing the crack data is needed for the smart film to monitor concrete structures. According to the characteristics of the remote monitoring, the system is divided into two parts, that is, the local and the remote monitoring center (see Figure 9).

The hardware of the system is mainly composed of smart film crack sensors, communication lines, power managements, wireless transmission equipments, industrial personal computer (IPC), the protection devices of power supply, and the server of the remote monitoring center. Here GPRS-DTU is used as the wireless transmission equipment. Hence the server of the remote monitoring center must have access to the Internet.

**Figure 10:**
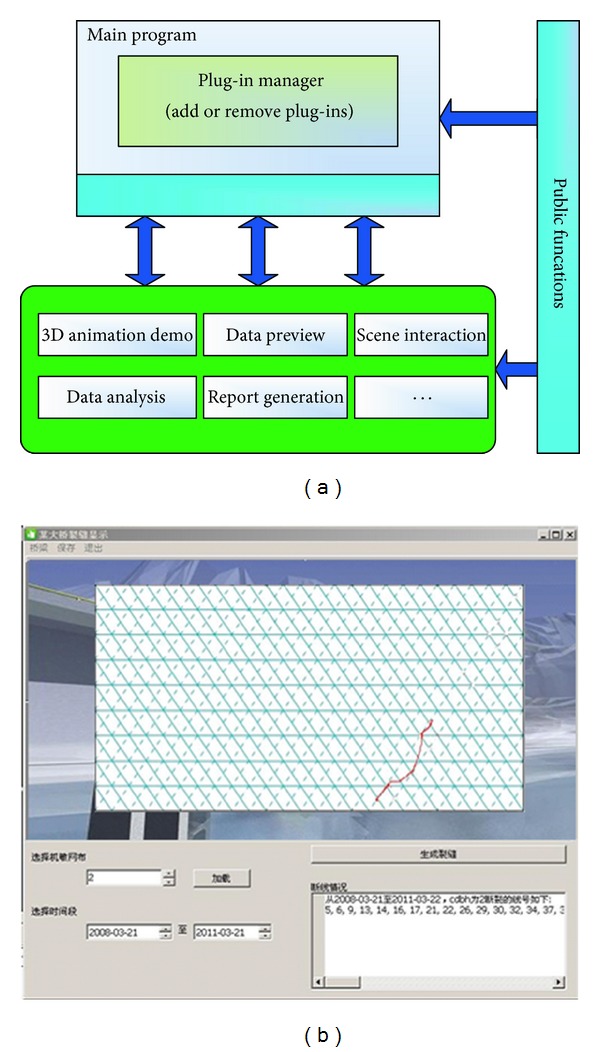
3D visualization system: (a) system framework and (b) the plug-ins of analysis and recurrence of crack data.

The remote monitoring center is based on the plug-in 3D visualization design, including database management, scene interaction, data analysis and recurrence, 3D animation design in structure, report generation, and interface design. To deal with the problem of data analysis and recurrence, the least squares B-spline curve algorithm, which is usually used for drawing curve from points, is adopted to compose the broken points into a virtual crack. Then, the readability of the data can be improved.  Figure 10 illustrates the 3D system.

## 5. Applications on Real Bridges

### 5.1. Masangxi Yangtze River Bridge

Masangxi Yangtze River Bridge, located in Dadukou district of Chongqing municipality, China, is a prestressed concrete cable-stayed bridge with the main bridge of 179 m + 360 m + 179 m. Its total length is 1104.23 m and its deck's width is 30.6 m. It is divided into two independent bridges and consists of a west platform at one end, 9 approach bridges at the other end. and a main bridge between them. The main bridge and the approach bridge have 12 spans. The west platform has a length of 5 m. One of the approach bridges is 44 m and the others are 40 m. The main bridge has the structure of double-tower and double-cable-plane, the section of the girder has the structure of prestressed concrete separate triangle-box, and a half cubic curve camber is sited between middle span and side span.

#### 5.1.1. Sensor Distribution

Before the installation of the monitoring system, a large number of cracks have existed in the Masangxi Yangtze River Bridge, and reinforcement of the durability and the structure has been implemented on two main towers according to the cracked condition. In order to ensure the safe operation of the bridge, the bridge crack monitoring system is installed on two towers, monitoring cracks including new and preexisting ones in real-time.

**Figure 11:**
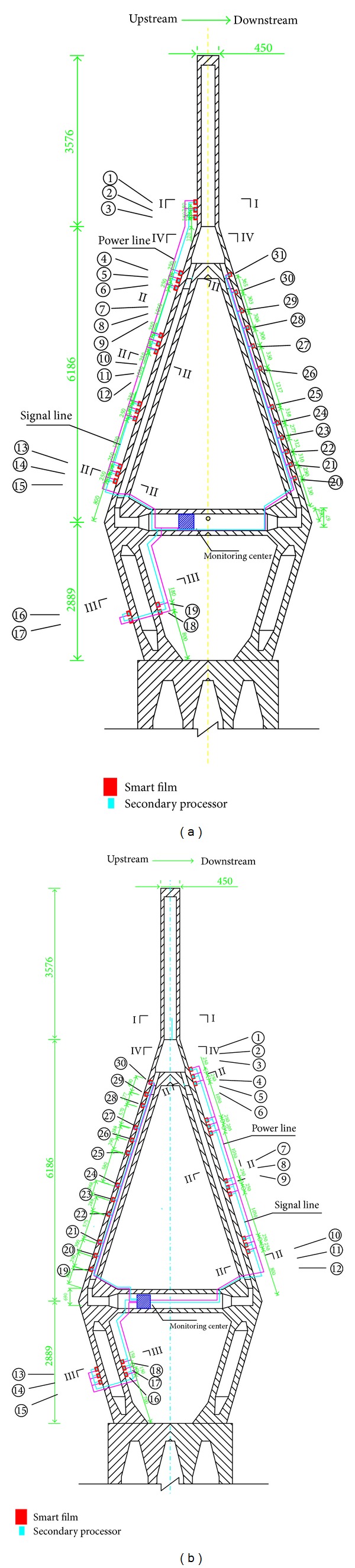
Illustration of sensor installation of tower crack monitoring system: (a) layout of the smart film and the secondary processor in tower no. 1 and (b) layout of the smart film and the secondary processor in tower no. 2.

For the upstream side of tower no. 1, 3 pieces of smart films (no. 1–no. 3) are pasted on the connection part between the upper part and the middle part of the tower, 12 pieces of films (no. 4–no. 15) on the middle part, and 4 pieces of films (no. 16–no. 19) on the lower part. For the downstream side of the tower, 12 pieces of smart films (no. 20–no. 31) are pasted on the inner part of the middle tower, as shown in Figure 11(a).

For the downstream side of tower no. 2, 12 pieces of smart films (no. 1–no. 12) are pasted on the middle part of the tower. For the upstream side of the tower, 6 pieces of smart films (no. 13–no. 18) are pasted on the lower tower and 12 pieces of films (no. 19–no. 30) on the inner part of the middle tower, as shown in Figure 11(b). According to the force condition and the pre-existing cracks of the tower, it is found that the cracks are mainly vertical. Therefore, the pasted smart films are only transverse; the smart film on the outer surface of the tower consists of 12 transverse enameled wires, but the film on the inner tower consists of 15 transverse wires.

#### 5.1.2. The Interactive 3D Scene

**Figure 12:**
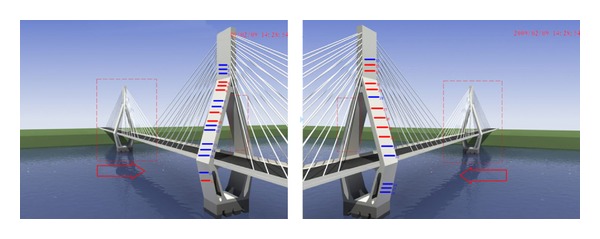
3D scene of Masangxi Yangtze River Bridge.

Since the 3D scene of Masangxi Yangtze River Bridge is formulated using 3DS MAX, the real geographic environment, bridge structure, and the distribution of the monitoring equipments can be simulated visually. After the running of plug-in of the interactive 3D scene, a single tower is used as the entrance and the scene interacts down layer by layer. In the main screen of each tower, a button of each smart film is created from different perspectives. When clicking the button, the crack will be recurred by the smart film (see Figure 12).

#### 5.1.3. Analysis and Recurrence of Crack Data

The monitoring time is from March 2008 to November 2009. During this period, it can be found that part of cracks (new and old ones) spread to a certain degree according to analysis of the collected data through the server of the remote monitoring center.

In tower no. 1, cracks were found in December 2008, which appeared on the smart films with sequence number of 3, 4, 6, 7, 9, 10, 24, 26, 29, 30, and 31. In addition, all the wires in the smart films with the sequence numbers of 4, 6, 9, 10, and 26 broke due to the penetration of cracks. The width of the smart film is 30 cm. Therefore, the lengths of the cracks are more than 30 cm.

**Figure 13:**
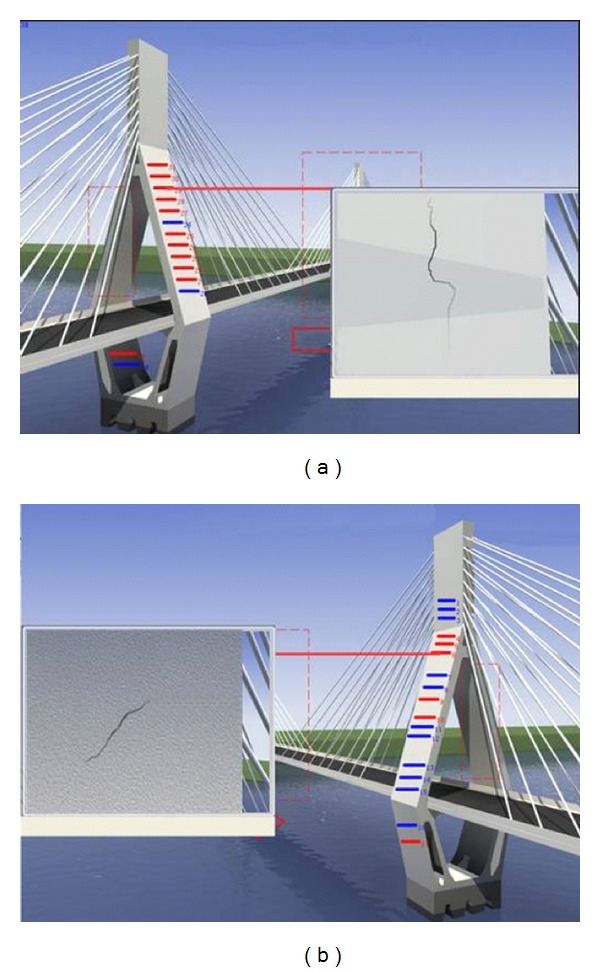
Visual recurrence of the real crack: (a) 29th smart film of tower no. 1 and (b) 31st smart film of tower no. 1.

According to data analysis, it can be known that: during December 2008, the breakage ratio of sensor 24 was 27%, the ratio of sensor 26 was 87%, the ratio of sensor 27 was 47%, and the ratio of sensor 29 was 27%. Till November 2009, the newly added breakage ratio of sensor 24 was 73% and the total breakage ratio was 100%. The newly added ratio of sensor 26 was 13% and the total ratio was 100%. The newly added ratio of sensor 27 was 13% and the total ratio was 60%. The newly added ratio of sensor 29 was 40% and the total ratio was 67%. Hence, during December 2008 and November 2009, the cracks on the locations of the smart films with the sequence numbers of 24, 26, 27, and 29 continued to extend, and the cracks pierced through the whole smart films with the sequence numbers of 24 and 26.  Figure 13 shows the visual recurrence of the real crack in 29th and 31st smart film of tower no. 1.  Table 1 presents data of the smart films of tower no. 1.

In tower no. 2, cracks were also found in December 2008, which appeared on the smart films with the sequence numbers of 4, 5, 6, 9, 17, 19, 21, 22, 23, 24, 25, 27, 28, 29, and 30. In addition, all the wires in the smart films with the sequence numbers of 6, 17, 19, 21, and 27 broke due to the penetration of cracks. Similarly, the lengths of the cracks are more than 30 cm.

**Figure 14:**
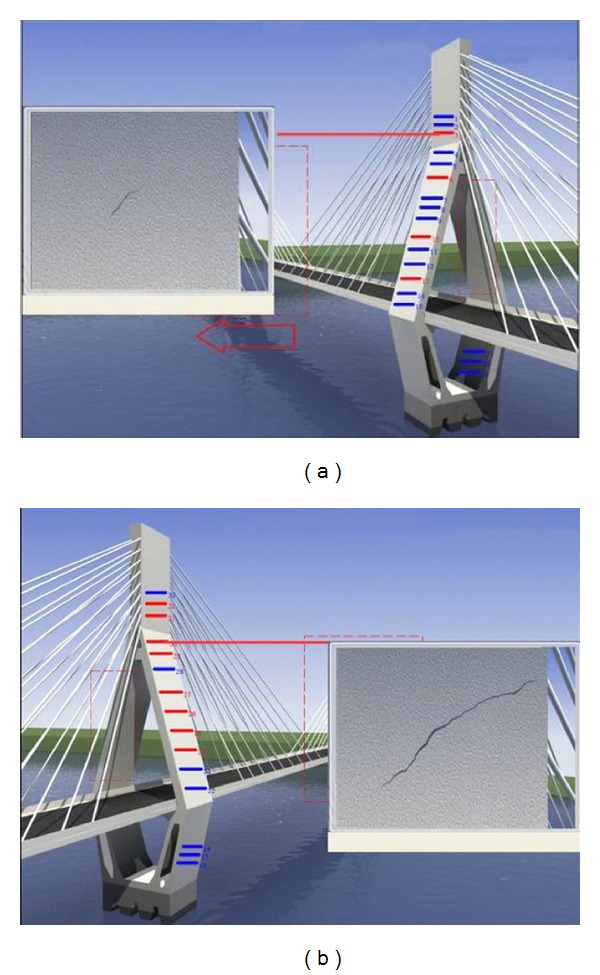
Visual recurrence of the real crack: (a) 4th smart film of tower no. 2 and (b) 30th smart film of tower no. 2.

According to data analysis, it can be known that, during December 2008, the breakage ratio of sensor 4 was 8%, the ratio of sensor 5 was 17%, the ratio of sensor 9 was 83%, the ratio of sensor 22 was 66%, the ratio of sensor 23 was 87%, the ratio of sensor 24 was 80%, the ratio of sensor 25 was 40%, the ratio of sensor 28 was 53%, the ratio of sensor 29 was 87%, the ratio of sensor 30 was 60%, and the ratio of sensor 31 was 73%. Till November 2009, the newly added breakage ratio of sensor 4 was 75% and the total breakage ratio was 83%. The newly added ratio of sensor 5 was 67% and the total ratio was 84%. The newly added ratio of sensor 9 was 17% and the total ratio was 100%. The newly added ratio of sensor 22 was 27% and the total ratio was 93%. The newly added ratio of sensor 24 was 7% and the total ratio was 87%. The newly added ratio of sensor 25 was 26% and the total ratio was 66%. The newly added ratio of sensor 28 was 47% and the total ratio was 100%. The newly added ratio of sensor 29 was 13% and the total ratio was 100%. The newly added ratio of sensor 30 was 40% and the total ratio was 100%. The newly added ratio of sensor 31 was 27% and the total ratio was 100%. Hence, during December 2008 and November 2009, the cracks on the locations of the smart films with the sequence numbers of 4, 5, 9, 22, 24, 25, 28, 29, 30, and 31 continued to extend, and the cracks pierced through the whole smart films with the sequence numbers of 9, 28, 29, 30, and 31. The crack on the location of the smart film with the sequence number of 23 did not extend.  Figure 14 shows the visual recurrence of the real crack in 4th and 30th smart film of tower no. 2.  Table 2 presents data of the smart films of tower no. 2.

#### 5.1.4. Comparison of Data

**Figure 15:**
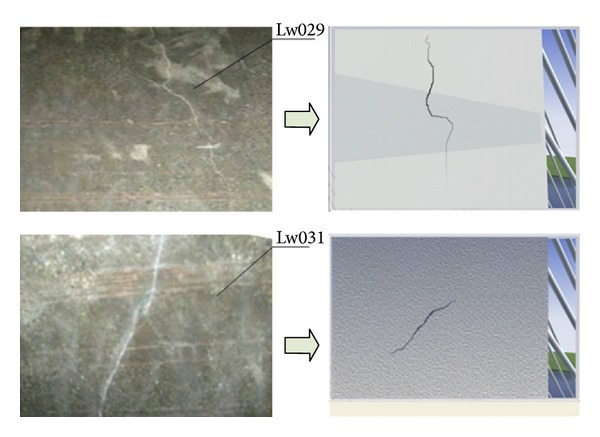
Comparison of tower no. 1 between man-operated detection and monitoring using the plug-in of recurrence of crack.

In order to verify the validity of monitoring data and grasp the propagation conditions of the cracks, professionals had been repeatedly sent to the scene to observe the problem.  Figure 15 shows the comparison charts of tower no. 1 between man-operated detection and monitoring using the plug-in of recurrence of crack. And it can be drawn that the result of remote recurrence of crack is basically consistent with that of the local detection.

#### 5.1.5. Monitoring Result

Before December 2008, new cracks were not found. And new cracks were not detected by the local detections of the professionals, especially after the Wenchuan earthquake in May. During December 2008, it could be found by the system that the structural cracks came to emerge. During December 2008 and October 2009, the structural cracks continued to extend. According to analysis of breakage time of the enameled wires, the results showed that the propagation of cracks on the towers experienced the process of relative stability, crack propagation, and relative stability. Currently, some cracks tend to extend, but in a relatively slow speed.

### 5.2. Tukan Wujiang River Bridge

Tukan Wujiang River Bridge, located in Yu-Xiang expressway connecting Chongqing Municipality and Hunan Province, China, is a prestressed continuous steel bridge with the main bridge of 110 m + 200 m + 110 m. It is divided into two independent bridges (the right bridge and the left bridge). The right bridge consists of a T-beam at one end, three T-beams at the other end, and a main bridge between them. The left bridge consists of the main bridge and three T-beams. The length of a T-beam is 30 m. A single cell and single box section is used in the box girder of the main bridge, whose width of roof is 12 m, width of base plate is 6 m, and width of unilateral cantilever is 3 m.

#### 5.2.1. Sensor Distribution

**Figure 16:**
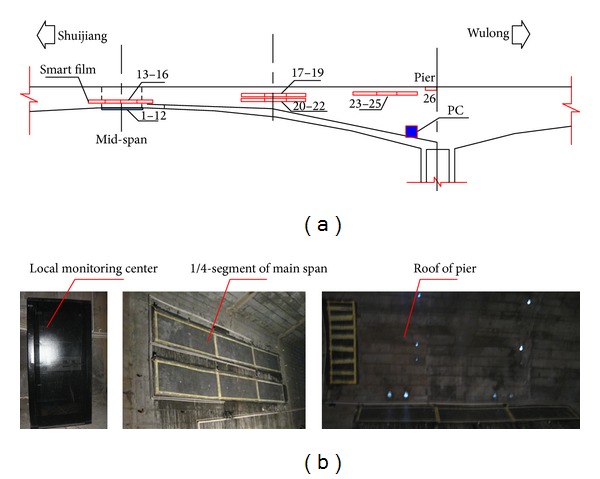
(a) Layout of smart film sensors and (b) the local monitoring center and smart film sensors.

The smart films are mainly pasted on pier, 1/4-segment of main span, the mid-segment of main span (the closure segment), which is shown in Figure 16. 26 pieces of smart films that have 80 enameled wires are pasted on each bridge. 3 pieces of films (no. 23–no. 25) are pasted on the plate of box girder of pier and 1 piece of film (no. 26) on the roof of box girder of pier. Six pieces of films (no. 17–no. 22) are pasted on the plate of box girder from 1/4-segment of main span. Four pieces of films (no. 13–no. 16) are pasted on the plate of box girder from the mid-segment of main span. 12 pieces of films (no. 1–no. 12) are pasted on the base plate of box girder from the mid-segment of main span. The smart films on the two bridges are symmetric with each other.

#### 5.2.2. The Interactive 3D Scene

**Figure 17:**
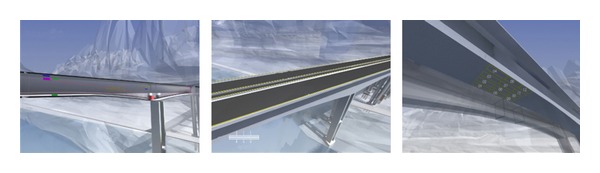
Effect of the interactive 3D scene.

Since the 3D scene of Tukan Wujiang River Bridge is formulated using 3DS MAX, the real geographic environment, bridge structure, and the distribution of the monitoring equipments can be simulated visually. After running of the plug-in of the interactive 3D scene, a single tower is used as the entrance and the scene interacts down layer by layer. In the main screen of each tower, buttons of each smart film are created from different perspectives. When clicking the buttons, the corresponding scenes will be recurred (see Figure 17).

#### 5.2.3. Analysis and Recurrence of Crack Data

The monitoring begins with December 2008, and the system runs normally until now. It can be found from data collected by the server of the remote monitoring center that some cracks emerged on the two bridges.

**Figure 18:**
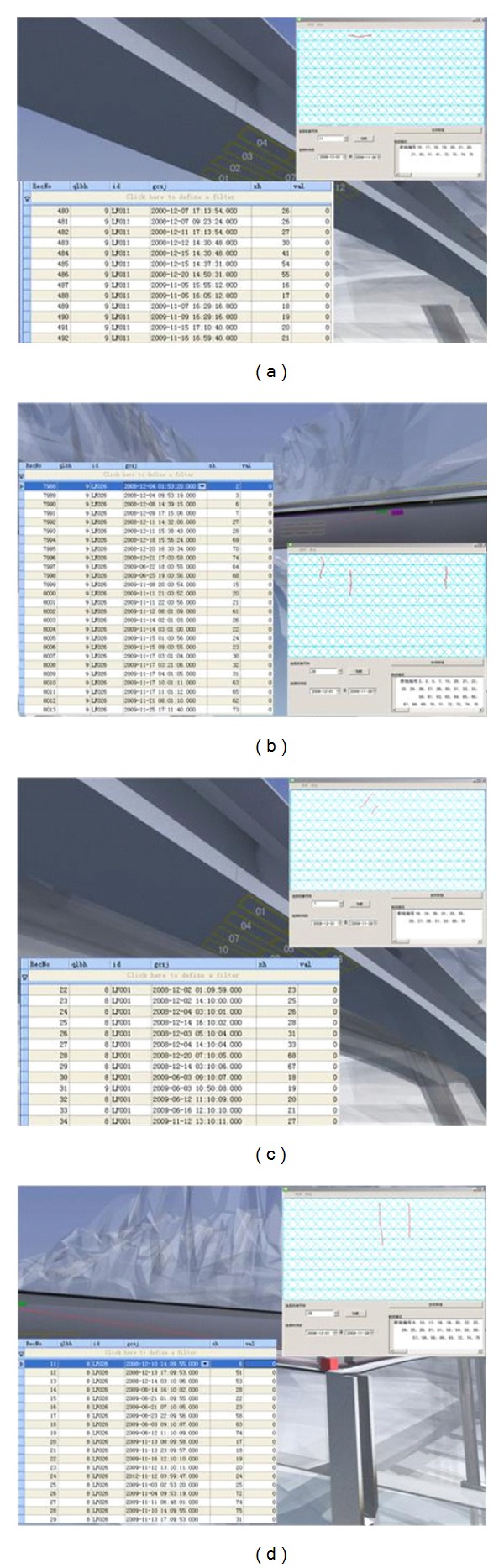
Recurrence of crack on Tukan Wujiang River Bridge.

Through the plug-in of analysis and recurrence of crack data, a vertical crack with a length about 15 cm could be found by sensor 11 (Lw011) which was arranged in the base plate of box girder from the mid-segment of main span of the right bridge, and it emerged in early November 2009, as shown in Figure 18(a). Three vertical cracks with the lengths ranging from 13 cm to 28 cm could be found by sensor 26 which was arranged close to the roof of box girder of pier 0 on the right bridge, and they appeared in early September 2009, as shown in Figure 18(b). Two vertical cracks with the lengths from 7 cm to 15 cm could be found by sensor 1 (Lw001) which was arranged in the base plate of box girder from the mid-segment of main span of the left bridge, and they occurred in mid February 2009, as shown in Figure 18(c). Two vertical cracks with the lengths from 24 cm to 35 cm could be found by sensor 26 (Lw026) which was arranged in the roof of box girder of pier 0 on the left bridge, and their initiation time was mid November 2009, as shown in Figure 18(d).

#### 5.2.4. Comparison of Data

**Figure 19:**
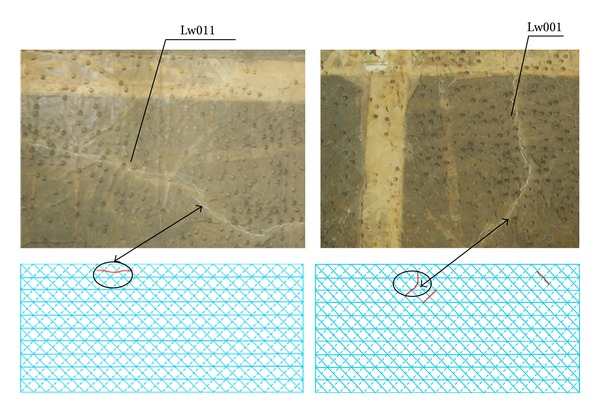
Data comparison.

During the monitoring period, the result of the plug-in of recurrence of crack was compared with that of man-operated periodic detection (see Figure 19). The locations which needed to be compared were Lw011 of the mid-segment of main span on the right bridge and Lw001 of the mid-segment of main span on the left bridge. The man-operated detection data is 16.8 cm and 12.3 cm, respectively. And the data is basically consistent with the result of the remote monitoring center.

#### 5.2.5. Monitoring Result

According to the data of the enameled wires of the smart film from February 2009 to November 2010, it can be inferred that several vertical cracks exist on the base plate of the mid-segment of main span and close to the base plate of box girder of pier. On the basis of the locations and the directions, these cracks mostly belong to nonstructural cracks due to concrete shrinkage and temperature variation.

Moreover, through the load test during the completion period (September 1-2, 2009), a few enameled wires of the smart films on the base plate of the mid-segment of main span were found broken for a short time then returned to normal later. This phenomenon could be explained as follows. During the load test period, force was loaded on the mid-segment of main span temporarily which led to the appearance of cracks, and then the wires were broken. When the load was released, crack would disappear and the wires went back into contact. This phenomenon could not be found by man-operated detection. In summary, no obvious structural cracks had been found on Tukan Wujiang River Bridge yet.

### 5.3. The Other Bridges

At present, the bridges equipped with the remote monitoring system based on smart film include Jiangjin Yangtze River Highway Bridge, Lijiatuo Yangtze River Bridge, Niupeng Bridge, and Heichonggou Bridge.

**Figure 20:**
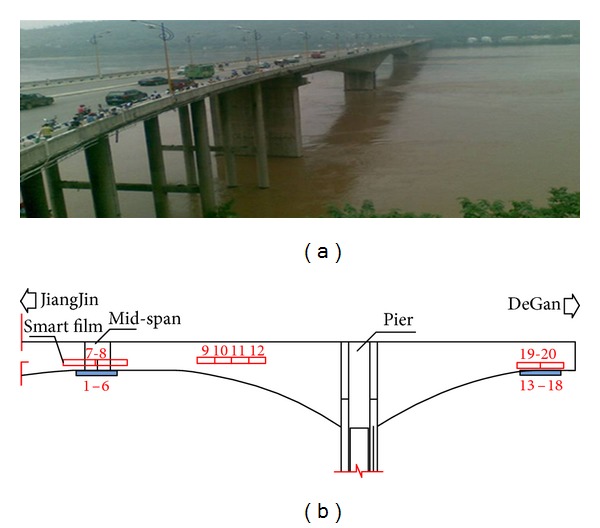
Jiangjin Yangtze River Highway Bridge and its sensor layout.

Jiangjin Yangtze River Highway Bridge, a prestressed continuous steel bridge with the main bridge 140 m + 240 m + 140 m, is located in Chongqing municipality, China. Its total length is 1360 m. A single cell and single box section is used in the box girder of the main bridge. The smart films are fabricated into crack sensors, which are installed on the mid-segment of main span, 1/4-segment of main span, and side span in Degan direction, respectively. The monitoring system was installed in April 2012, and it is still in use now (see Figure 20).

**Figure 21:**
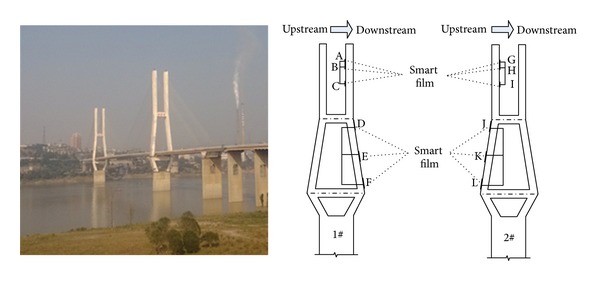
Lijiatuo Yangtze River Bridge and its sensor layout.

Lijiatuo Yangtze River Bridge, located in Chongqing Municipality, China, is a prestressed concrete cable-stayed bridge with the main bridge of 169 m + 444 m + 169 m. Its total length is 1288 m. The bridge has a structure of double-tower and double-cable-plane and consists of two transitional holes, eight approach bridges, and a main bridge. Each transitional hole has a length of 53 m. The length of each approach bridge is 50 m. Two sets of smart film crack monitoring systems are installed on two towers, respectively. For each tower, sensors are installed on two connection parts. For the girder, sensors are installed on 1/4-segment of two side spans, the mid-segment of main span, and 1/4-segment of main span. The monitoring system was installed in September 2012. So far, the systems are running well (see Figure 21).

**Figure 22:**
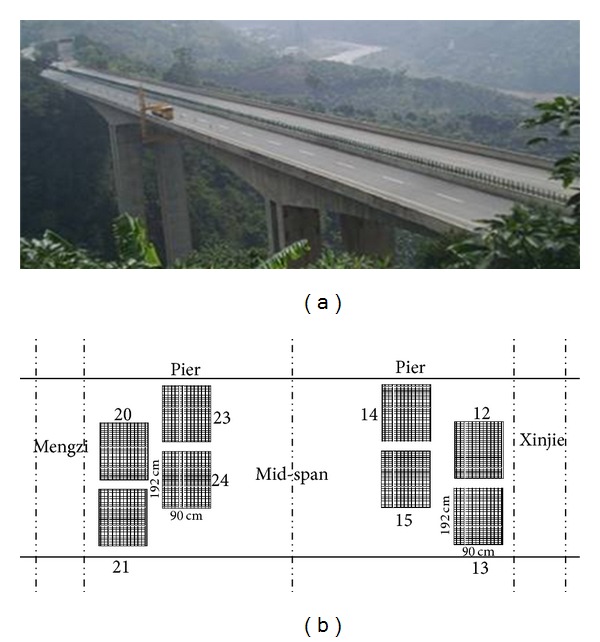
Niupeng Bridge and its sensor layout.

Niupeng Bridge, located in Lvshui River area of Meng-Xin expressway connecting Mengzi and Xinjie, Yunnan province, China, is into a continuous steel bridge with the main bridge of 77 m + 140 m + 77 m. Its total length is 365 m. It is divided two independent bridges. A single cell and single box section is used in the box girder of the main bridge. Crack sensors are installed on the roof of pier of Mengzi Direction, the roof of pier of Xinjie Direction, and the mid-segment of main span. The monitoring system was installed in December 2012 and is still in use until now (see Figure 22).

**Figure 23:**
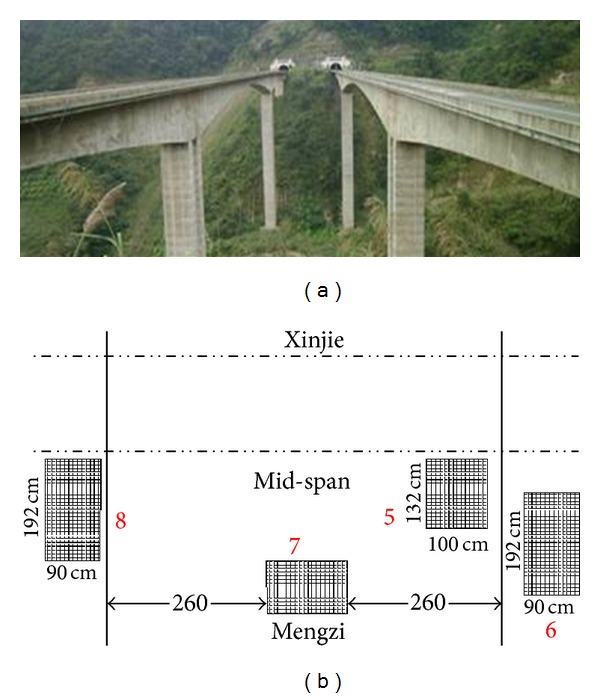
Heichonggou Bridge and its sensor layout.

Heichonggou Bridge, located in Zhamakong of Meng-Xin expressway connecting Mengzi and Xinjie, Yunnan province, China, is into a prestressed concrete continuous bridge with the main bridge of 98 m + 180 m + 98 m. It is divided two independent bridges. A single cell and single box section is used in the box girder of the main bridge. Crack sensors are installed on the roof of pier of Mengzi Direction, the roof of pier of Xinjie Direction, the mid-segment of main span, and plate of 1/8-span of the left bridge. The monitoring system was installed in December 2012. So far, the system is running normally (see Figure 23).

As discussed above, apart from the unique damage location due to bridge crack, sensors are mainly installed on the upper part of the tower, the middle part, the lower part, and two connection parts of the tower for the cable-stayed bridge. For the main girder, sensors are mainly installed on the locations where cracks are easy to initiate, including the base plate and the plate of the main bridge as well as the plate of 1/4-segment of main span. For the continuous steel bridge, sensors are mainly installed on the following locations: the base plate and the plate of the mid-segment of main span, the plate of 1/4-segment of main span, the plate of 1/8-segment of main span, the roof of pier, the base plate, and the plate of the closure segment in side span.

## 6. Conclusion

Smart film crack monitoring method, proposed by the authors, is a good choice for detecting initiation, length, width, shape, location, and propagation of cracks on concrete bridges. In this paper, a methodological approach on bridge crack monitoring with smart film has been developed. Firstly, this paper examines a new fabrication technique of the smart film based on structural characteristics which overcomes the difficulties found in other measures. Then the feasibility of crack monitoring is analyzed and verified by the mechanical sensing character of the smart film under the two conditions of normal strain and crack initiation. The result shows a good sensing performance under the two conditions. At the same time, the coupling effect between parallel enameled wires of the smart film is discussed, and then it is reduced by use of low-frequency detecting signal along with the custom communication protocol. On this basis, the remote crack monitoring system with smart film, consisting of the local monitoring center and the remote monitoring center, is designed. Then crack data collected by the local monitoring center is sent to the remote monitoring center and the cracks are simulated and recurred by the plug-ins of the interactive 3D scene and analysis of crack data, improving the readability of data.

Finally, the monitoring system has been applied to six bridges, including Masangxi Yangtze River Bridge, Tukan Wujiang River Bridge, Jiangjin Yangtze River Bridge, Lijiatuo Yangtze River Bridge, Niupeng Bridge, and Heichonggou Bridge. The monitoring effect has been discussed. The monitoring conditions on Masangxi Yangtze River Bridge and Tukan Wujiang River Bridge are demonstrated in detail. At the same time, the accuracy of the monitoring result is verified by the comparison between man-operated periodic detection and the system monitoring. Through the long-term operation of the system, the system concludes with a good stability (the running time of the system on Tukan Wujiang River Bridge is over 5 years), reliability, and instantaneity. It is useful for evaluation of structural security of bridges.

## Figures and Tables

**Table 1:** Data of the smart films of tower no. 1.

Sensor number	3	4	6	7	9	10
The total breakage ratio during December 2008	42%	100%	100%	92%	100%	100%
The total breakage ratio during November 2009	42%	100%	100%	92%	100%	100%
Sensor number	24	26	27	29	30	31
The total breakage ratio during December 2008	27%	87%	47%	27%	60%	67%
The total breakage ratio during November 2009	100%	100%	60%	67%	60%	67%

**Table 2:** Data of the smart films of tower no. 2.

Sensor number	4	5	6	9	17	19	21	22
The total breakage ratio during December 2008	8%	17%	100%	83%	100%	100%	100%	66%
The total breakage ratio during November 2009	83%	84%	100%	100%	100%	100%	100%	93%
Sensor number	23	24	25	27	28	29	30	31
The total breakage ratio during December 2008	87%	80%	40%	100%	53%	87%	60%	73%
The total breakage ratio during November 2009	87%	87%	66%	100%	100%	100%	100%	100%
